# The enzymatic hydrolysis of pretreated pulp fibers predominantly involves “peeling/erosion” modes of action

**DOI:** 10.1186/1754-6834-7-87

**Published:** 2014-06-10

**Authors:** Valdeir Arantes, Keith Gourlay, Jack N Saddler

**Affiliations:** 1 University of British Columbia, 2424 Main Mall, Vancouver, BC V6T 1Z4, Canada

**Keywords:** Biomass deconstruction, Cellulose hydrolysis, Mechanism of enzymatic deconstruction

## Abstract

**Background:**

There is still considerable debate regarding the actual mechanism by which a “cellulase mixture” deconstructs cellulosic materials, with accessibility to the substrate at the microscopic level being one of the major restrictions that limits fast, complete cellulose hydrolysis. In the work reported here we tried to determine the predominant mode of action, at the fiber level, of how a cellulase mixture deconstructs pretreated softwood and hardwood pulp fibers. Quantitative changes in the pulp fibers derived from different pretreated biomass substrates were monitored throughout the course of enzymatic hydrolysis to see if the dominant mechanisms involved either the fragmentation/cutting of longer fibers to shorter fibers or their “peeling/delamination/erosion,” or if both cutting and peeling mechanisms occurred simultaneously.

**Results:**

Regardless of the source of biomass, the type of pretreatment and the chemical composition of the substrate, under typical hydrolysis conditions (50°C, pH 4.8, mixing) longer pulp fibers (fiber length >200 μm) were rapidly broken down until a relatively constant fiber length of 130 to 160 μm was reached. In contrast, shorter fibers with an initial average fiber length of 130 to 160 μm showed no significant change in length despite their substantial hydrolysis. The fragmentation/cutting mode of deconstruction was only observed on longer fibers at early stages of hydrolysis. Although the fiber fragmentation mode of deconstruction was not greatly influenced by enzyme loading, it was significantly inhibited by glucose and was mainly observed during initial mixing of the enzyme and substrate. In contrast, significant changes in the fiber width occurred throughout the course of hydrolysis for all of the substrates, suggesting that fiber width may limit the rate and extent of cellulose hydrolysis.

**Conclusion:**

It appears that, at the fiber level, pretreated pulp fibers are hydrolyzed through a two-step mode of action involving an initial rapid fragmentation followed by simultaneous swelling and peeling/erosion of the fragmented fibers. This latter mechanism is the predominant mode of action involved in effectively hydrolyzing the cellulose present in pretreated wood substrates.

## Background

Monomeric sugars derived from cellulosic materials hold great potential as abundant, renewable intermediates for the production of renewable liquid transportation fuels, chemicals, and biomaterials. However, despite its supposedly simple chemical composition, consisting only of D-glucopyranosyl residues linked by beta-1,4-glycosidic bonds, the cellulose in higher plants exists as highly ordered and tightly packed insoluble microfibrils that are intrinsically associated with other non-cellulosic moieties (such as lignin and hemicellulose), resulting in complex and heterogeneous morphologies that are recalcitrant to enzymatic hydrolysis. The diversity, heterogeneity, and complex morphology of cellulosic biomass all contribute to its recalcitrance, and in turn, to our current limited ability to achieve rapid and near complete breakdown of cellulosic substrates at low enzyme loading. Even after the significant improvements that the enzyme companies have made in increasing the specific activity of “cellulase mixtures,” about a one to two orders of magnitude higher protein loading is required to break down cellulose as compared to starch [1].

Despite the discovery and addition of auxiliary enzymes and proteins such as lytic polysaccharide monooxygenase and Swollenin, effective cellulose hydrolysis is still primarily achieved by the cooperative interaction of beta-1,4-endoglucanases (shown to randomly cleave accessible beta-1,4-glycosidic bonds, releasing soluble cellodextrins and creating new free chain ends on the cellulose surface), beta-1,4-exoglucanases/cellobiohydrolases (which hydrolyze cellulose processively from the chain ends releasing mostly cellobiose), and beta-glucosidases (which hydrolyse cellodextrins, including cellobiose, to glucose) [2].

However, there is still considerable debate regarding the actual mechanisms by which a “cellulase mixture” deconstructs the cellulosic component of pretreated biomass substrates, particularly at the fiber, fibril, and microfibril levels of plant cellular structure [3]. Some workers using “model/pure” cellulose substrates have suggested that the initial mode of enzymatic attack takes place on the outer layer of the cellulose surface where the constituent fibers are peeled along their length, layer by layer, in an “onion peeling” fashion [4-8]. This peeling mode of action has also been termed a “shaving” or “planing” mode of action. Alternatively, it has been suggested that cellulose deconstruction is a two-step process where the cellulose-rich fibers are initially fragmented or disaggregated into shorter fibers, resulting in a greater overall surface area for the enzymes to subsequently attack [9-12]. This “cutting” mode of deconstruction has also been termed a “fragmentation” or “scissoring” mode of action. Other work that has used “model/pure” cellulosic substrates has suggested [13,14] that the dominant mode of deconstruction was strongly influenced by the fiber dimensions of the initial substrate, with the larger particles first fragmented/cut into smaller particles (“cutting”) while the smaller particles seemed to be hydrolyzed by a “peeling/erosion” type of mechanism.

These sometimes contradictory observations and conclusions on the mechanism of enzymatic attack at the fiber level were likely caused by the many variables involved in the experimental design, ranging from differences in the cellulosic substrates that were used, to differences in the individual and combinations of enzymes that were employed. In the majority of these previous studies, model cellulosic substrates such as Avicel, Solka Floc, filter paper, bacterial cellulose, and cotton were used, primarily to try to minimize the influence that other biomass components, such as lignin and hemicelluloses, might have on the interpretation of the results. However, a considerable amount of previous work has shown that lignin and hemicellulose play important roles in restricting enzyme access to the cellulose [15,16], and consequently they would also be expected to influence the performance of cellulases during the enzymatic hydrolysis of cellulose. To complicate the story further, other components of an effective “cellulase mixture” now include auxiliary enzymes such as xylanases, polysaccharide monooxygenases, and non-catalytic proteins such as amorphogenesis-inducing proteins (for example, Swollenin) [17,18]. This adds to the challenge of trying to better understand the mode of action of enzymes and the fiber characteristics that limit the effective hydrolysis of the cellulose component within pretreated biomass substrates.

In the work reported here, a library of SO_2_-catalyzed steam or ethanol-organosolv pretreated hardwood and softwood pulp fibers with varying physical (fiber length and width) and chemical (lignin, hemicellulose, and cellulose content) properties within the range of those likely to be found in the emerging biorefinery industry sector was created. These substrates were subsequently used to determine the predominant mode of action, at the fiber level, of how a commercial cellulase mixture might hydrolyze industrially relevant feedstocks. The predominant enzymatic mode of action at the fiber level was assessed by monitoring chemical and physical/morphological changes during hydrolysis. We also hoped to determine whether the dominant mechanism was substrate dependent and if the insights could be applied to enhance the effective hydrolysis of industrially relevant pretreated lignocellulosic substrates.

## Results and discussion

Although strategies such as batch, feed-batch, and two-stage hydrolysis have been evaluated on a range of pretreated substrates, the development of an efficient hydrolysis process has been limited, at least in part, by the lack of understanding of how commercially relevant pretreated pulp fibers are enzymatically hydrolyzed. Therefore, there is a need to better understand the enzymes’ mode of action, which seems to occur primarily at the fiber level. Unfortunately, many of the past efforts that have tried to elucidate the predominant modes of hydrolysis have been contradictory. Therefore, a range of pretreated pulp fiber fractions was first created in order to determine the mechanism by which a “cellulase mixture” actually hydrolyzes pretreated pulp fibers at the macromolecular level and how dependent the predominant mechanisms are on the type of substrate and its physicochemical characteristics.

It was anticipated that steam pretreatment would primarily result in solubilization of the hemicellulose component while organosolv would predominantly remove the lignin from each of the hardwood and softwood substrates (Table 1). As had been observed previously [19,20], the organosolv pretreated softwood had the lowest lignin content while the steam pretreated softwood had the highest. The glucan content ranged from 48 to 60% for the steam pretreated substrates, and up to about 75% for the organosolv pretreated sample (Table 1). As expected, xylan was the major hemicellulose carbohydrate detected in the hardwood (poplar) derived fractions and mannan in softwoods (Douglas fir and lodgepole pine) (Table 1). When the poplar wood chips were pretreated at two different severities (*T* = 180°C and *T* = 200°C), to try to generate substrates that differed in their xylan content (to see if the amount of residual hemicellulose might influence changes in the mode of action of enzymes during subsequent enzymatic hydrolysis), twice as much xylan was retained after the lower severity treatment.

**Table 1:** Pretreatment conditions and chemical composition of the pretreated substrates

**Substrate**	**Pretreatment conditions**	**Composition of pretreated feedstocks (%)**^**1**^						**Abbreviation**
*SO*_*2*_*-steam pretreatment*		**Ara**	**Gal**	**Glu**	**Xyl**	**Man**	**AIL**	
Douglas fir	195°C, 4.5 min, 4.5% SO_2_	0.4	1.1	48.2	0.9	2.1	47.2	SPDF
Poplar	180°C, 5 min, 3% SO_2_	0.3	0.8	59.8	6.6	1.3	30.4	SPP180
Poplar	200°C, 5 min, 3% SO_2_	0.3	0.8	59.3	3.72	1.2	33.9	SPP200
*Ethanol-organosolv pretreatment*								
Lodgepole pine	170°C, 60 min; 65% EtOH, 1.1% H_2_SO_4_	bdl	bdl	74.8	1.6	1.8	17.3	OPLP

^1^Ara, arabinan; Xyl, xylan; Glu, glucan; Gal, galactan; Man, mannan; AIL, acid insoluble lignin; *bdl* below detectable level.

As described in the Materials and Methods section, various fiber populations that differed in terms of their initial fiber distribution were obtained by sieving each of the steam and organosolv pretreated substrates using a set of Bauer-McNett sieves (20, 40, and 100 mesh), and recovering each of the major sieved fractions (on a weight basis). The arithmetic average fiber length and width of the major pretreated pulp fractions were measured using a Fiber Quality Analyzer (FQA). As both steam and organosolv pretreatments were performed under acid-catalyzed conditions, some fiber shortening occurred as compared to untreated biomass fibers, due to chemical cutting during pretreatment. The resulting major sieved pulp fibers were distributed over a broad range of fiber lengths, from approximately 150 μm to approximately 1,850 μm (Table 2). As expected, the steam pretreated pulp fiber fractions were significantly shorter than the organosolv pretreated pulp fractions, confirming the more pulping mode of pretreatment exemplified by organosolv where the primary mechanism is the solubilization and removal of the lignin fraction with retention of much of the fiber integrity. The steam pretreated substrates underwent significant mechanical disruption, resulting from the quick discharge (explosion) of the biomass substrate, resulting in significant fiber shortening. It was also apparent that the severity of the pretreatment used had a significant effect on the fiber length of the resulting pulp, as evidenced by the sharp drop in the average fiber length of the major fraction of steam pretreated poplar when the pretreatment temperature (higher severity) was increased from 180°C (SPP180 R20) to 200°C (SPP200 R40) (Table 2).

**Table 2:** Initial average fiber dimensions for pretreated woody substrates

**Sample**	**Fiber length (μm)**	**Fiber width (μm)**
SPP200 R20	426.0^(23.1)^	25.20^(0.42)^
SPP200 R40	316.5^(19.1)^	24.65^(0.63)^
SPP180 R20	764.0^(45.3)^	26.95^(0.36)^
SPDF R100	372.5^(9.2)^	29.55^(0.73)^
SPDF F100	148.0^(0.4)^	25.85^(0.49)^
OPLP R20	1853.5^(7.8)^	32.15^(0.22)^

Numbers in parentheses indicate the standard deviation.

Although there were significant differences in the fiber lengths of the various fractions (Table 2), the average initial fiber width values were not substantially different, with the SPP samples having very similar (approximately 25 μm) widths (Table 2). Thus, the SPP200 R20 and R40 fiber fractions were able to provide the same pretreated materials that differed in their initial fiber lengths but were similar in their fiber width and chemical composition.

As the limits of resolution of the FQA did not allow us to quantify fibers with lengths and widths smaller than 50 μm and 6 μm, respectively, we used scanning electron microscopy to confirm that only the SPDF F 100 fraction contained any particles that were less than 50 μm in length and 6 μm in width. The chemical composition of all the size-fractionated pulp fibers was very similar to that of the unfractionated pretreated substrates (data not shown). This was in agreement with the previous work of Mooney et al. [21] and Del Rio et al. [22], who found that size fractionation of steam and organosolv pretreated woody feedstocks, respectively, resulted in fractionated pulp fibers of a similar chemical composition to that of the unfractionated pulps.

### Enzymatic hydrolysis of size-fractionated pulp fibers

**Figure 1:**
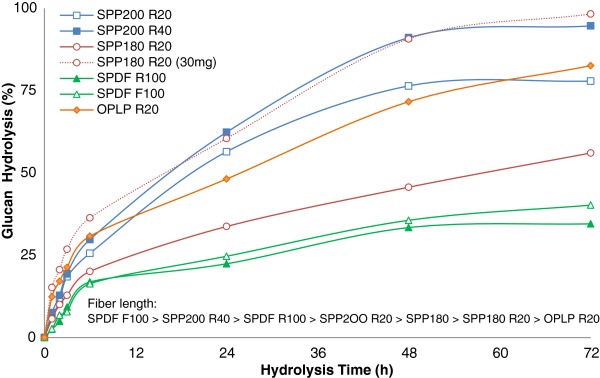
**Time-course hydrolysis of size-fractionated steam and organosolv pretreated pulp fibers.** Cellic CTec2 loading was 15 mg protein/g glucan unless labeled otherwise.

The six fractionated samples with average initial fiber length ranging from 150 to 1,850 μm were next enzymatically hydrolyzed at a protein loading of 15 mg/g glucan (Figure 1). After assessing several protein loadings (5 to 40 mg protein/g glucan), 15 mg/g glucan was selected as it provided the biggest differences in the hydrolysis yields of the fractionated pulp fibers (data not shown). In general, no clear correlation was observed between hydrolysis yields and the initial fiber length of the various size fractionated steam and organosolv pretreated softwood and hardwood substrates. This was not unexpected, as previous work [23] had shown that various substrates, including Douglas fir, poplar, and lodgepole pine, which had been subjected to the same or different pretreatment technologies, differed in their ease of hydrolysis and that this could not be explained solely by differences in their fiber lengths. When the initial rate of hydrolysis of the various pulp fractions from the same pretreated substrate such as SPP200 R20 (average initial fiber length of 426 μm) and SPP200 R40 (average initial fiber length of 317 μm), as well as SPDF R100 (average initial fiber length of 372 μm) and SPDF F100 (average initial fiber length of 148 μm) were compared, the initial fiber length did not seem to influence the initial rate of hydrolysis. To assess the possible influence of enzyme loading on the rate and extent of hydrolysis, the steam pretreated poplar SPP180 R20 fraction was also hydrolyzed at a twofold higher enzyme loading (30 mg/g glucan). As expected, the higher enzyme loading resulted in a substantial increase in both the rate and extent of hydrolysis (Figure 1).

### Changes in fiber properties during enzymatic hydrolysis

**Figure 2:**
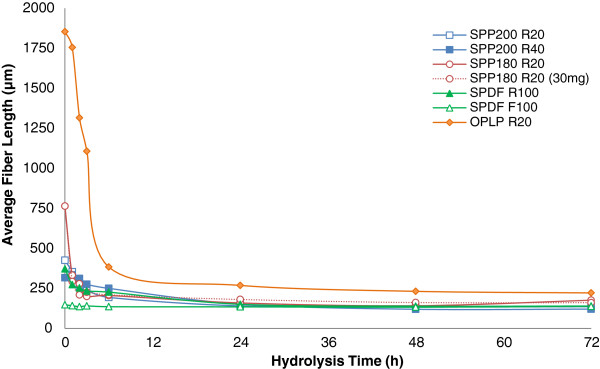
Changes in the average fiber length of the size-fractionated steam and organosolv pulp fibers during the course of enzymatic hydrolysis.

**Figure 3:**
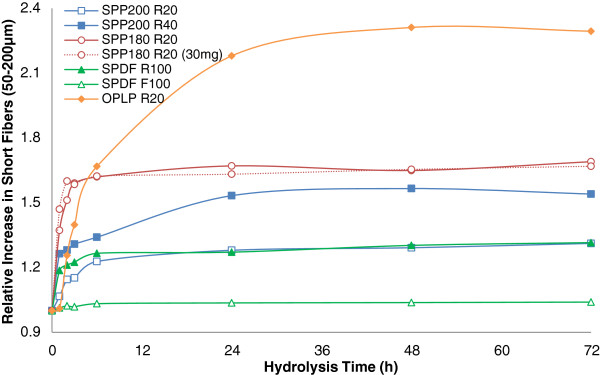
Relative changes in the population of short (100 to 200 μm) fibers over the course of hydrolysis of size-fractionated steam and organosolv pretreated pulp fibers relative to unhydrolyzed pulp fibers.

**Figure 4:**
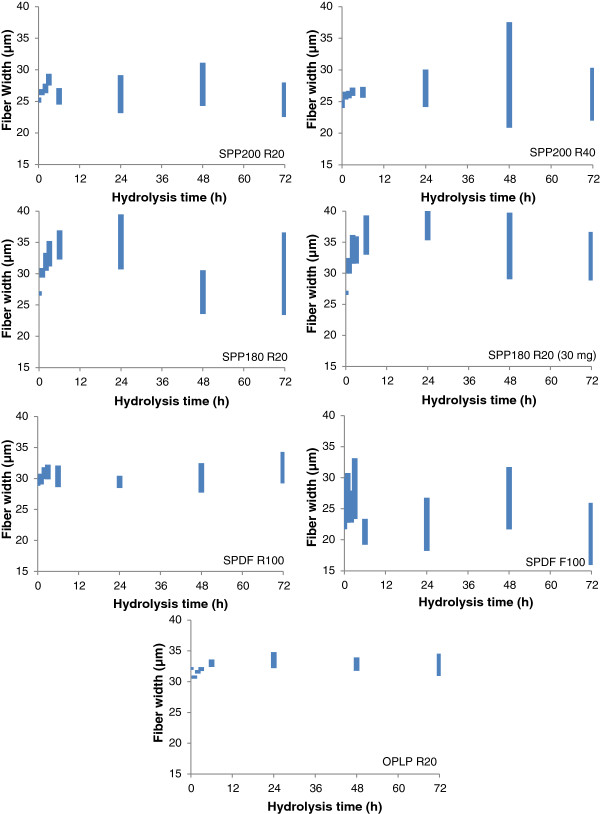
**Changes in the average fiber width of pretreated woody biomass over the course of hydrolysis of size-fractionated steam and organosolv pretreated pulp fibers.** The height of the bars represents the range/variability of the average fiber width in the sample (95% confidence interval).

To try to quantify any substrate changes occurring during the course of enzymatic hydrolysis, the partially hydrolyzed residues were also analyzed using the FQA to see if there were any changes in the average fiber length (Figure 2 and Figure 3) and fiber width (Figure 4) of the various fiber fractions.

Under typical hydrolysis conditions (50°C, pH about 4.8, with shaking/mixing), regardless of the chemical composition and pretreatment methodology and condition used, all of the fiber fractions were substantially fragmented within 1 to 3 h (Figure 2). The only exception was the SPDF195 F100 fraction, which had the shortest initial fiber length and displayed no change in the average fiber length over the course of hydrolysis. Interestingly, even though the lengths of the pulp fibers were reduced substantially within the first 3 h of hydrolysis at an enzyme loading of 15 mg/g glucan, the hydrolysis yields were quite low (<20%, Figure 1). Although fragmentation continued for up to 6 h for some fractions, in all cases the steam pretreated pulp fibers were fragmented until they reached a relatively constant value of about 130 to 160 μm, at which point there was a leveling off in the reduction of the length of the fibers. Thereafter, no further changes in fiber length were observed, even though most of the cellulose hydrolysis occurred during this period (6 to 72 h).

For the organosolv pretreated sample, OPLP R20, a further slight decrease in fiber length was observed between 6 to 24 h when the average fiber length was reduced to a relatively constant value of about 200 to 220 μm. This observation is in agreement with earlier work [22] which showed that various size-fractionated organosolv pretreated lodgepole pine samples with initial average fiber length higher than 200 and up to 3,400 μm were also rapidly fragmented. These authors also found that fiber fragmentation leveled off at a relatively constant value of 200 μm within the first 3 h of hydrolysis and that prolonged incubation resulted in little change in the average fiber length despite significant hydrolysis of the cellulose during this time [22].

Interestingly, the SPDF195 F100 fraction, with an average initial fiber length of 130 to 160 μm, which is similar to the leveling-off fiber length observed with the steam pretreated substrates, did not show any changes in its average fiber length, even during the initial few hours of hydrolysis.

Previously, Thygesen and colleagues [24] showed that, during the initial stages of hydrolysis of hydrothermally pretreated wheat straw, cellulase enzymes penetrate and cut the fibers at irregular regions along the lengths of the fiber wall known as dislocations. In related work, Clarke et al. [12] showed that lignin-free fibers were partially cut at dislocation sites during enzymatic hydrolysis. Based on these previous observations, it is likely that the longer fibers would have more dislocation sites along their surface when compared to the shorter fibers, accounting for the differences in extent of fragmentation between the different fiber fractions.Although a decrease in the average initial fiber length (fragmentation) probably resulted in an increase in the total number of fibers, it did not provide a measure of how the particle size distribution was changing with time. When assessing the particle distribution of short fibers for each sample, it appears that, at the early stages of hydrolysis, the number of short fibers in the range of 100 to 200 μm increased dramatically as a result of the fragmentation of the longer fibers (Figure 2 and Figure 3). The fiber fractions that did not show fiber fragmentation between 6 to 24 h (SPDF195 R100, SPP180 R20, SPDF195 F100, SPP200 R20), also did not show any change in the relative number of short fibers, although the SPP200 R40 fraction showed some fiber fragmentation between 6 to 24 h, which was followed by a relative increase in the number of short fibers.No significant change in fiber length was observed in the absence of enzymes (data not shown). However, it was apparent that the rate and extent of fragmentation as well as the pattern of the formation of short fibers (Figure 3) for steam pretreated poplar (SPP R20) at 15 and 30 mg protein/g glucan were similar (Figure 2), even though the hydrolysis rate and yields at 30 mg/g were considerably higher when compared to those at 15 mg/g (Figure 1).

The observed rapid reduction in fiber length occurs significantly prior to the slowdown in enzymatic hydrolysis that occurs toward the end of hydrolysis, usually when conversion levels reach approximately 70 to 80%. This implied that, although fiber fragmentation is one of the early macroscopic changes occurring during enzymatic hydrolysis, continuous fragmentation of the fiber does not seem to be required to maintain effective deconstruction throughout the course of hydrolysis. This in turn suggests that, after initial fragmentation, enzymatic hydrolysis occurs primarily in the width dimension.

For most of the fiber fractions, with the exception of the SPDF195 F100 fraction, the standard deviations for the mean fiber widths of the unhydrolyzed samples were very small (0.5 to 1.5 μm), indicating a high degree of uniformity of fiber width among the various size-fractionated and unfractionated samples (Table 2 and Figure 4).No correlation was observed between the rate and extent of hydrolysis and the changes in fiber width, other than changes in fiber width that were observed throughout the course of hydrolysis (Figure 4), in parallel with glucose release (Figure 1). Changes in fiber width occurred throughout the course of hydrolysis regardless of the source of biomass, the type and severity of pretreatment used, and the chemical composition of the pulp fibers. Interestingly, all of the substrates exhibited a substantial increase (up to 20 μm, equivalent to an 80% increase) in the variability of their mean fiber width. This suggests that, although the average fiber width itself does not seem to change substantially during hydrolysis, the numbers of fibers with widths much larger than average, as well as the number of fibers with widths much smaller than average, increased dramatically during hydrolysis. The increase in the standard deviation associated with fiber width became more pronounced after the first 6 h of hydrolysis, around the same time that the initial fiber fragmentation process was leveling off. Together, these results suggest that enzymatic deconstruction of pretreated lignocellulosic substrates occurs through an initial reduction in fiber length, followed by enzymatic activity promoting both fiber swelling and reductions in fiber width.

It is possible that most of the hydrolysis proceeds at or in between the layers of the cell wall, resulting in simultaneous swelling and peeling of the fiber during hydrolysis, rather than proceeding from each end of the fiber fragment. These two processes (swelling/peeling) would likely have a contrary influence on any changes occurring in fiber width, (swelling increasing the width while peeling would reduce it) possibly accounting for the observed variability in the fiber width values.For example, for all of the hardwood fiber fractions, a slight increase in the mean fiber width was observed within the first few hours of hydrolysis (Figure 4), suggesting that the swelling process might have proceeded faster than the peeling process.

### Effect of shaking and soluble sugar on fiber properties and hydrolysis yields

**Figure 5:**
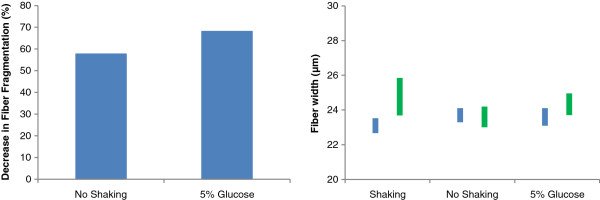
**The influence of shaking and glucose on fiber fragmentation and changes in fiber width during hydrolysis of size-fractionated pulp fibers SPP200 R20 at 15 mg/g glucan for 6 h at 2% solids loading (w/w).** Left) Decrease in the extent of fiber fragmentation; right) change in the average fiber width represented as changes in the range/variability of the average fiber width in the sample (95% confidence interval). Blue bar: control sample (only substrate); green bar: 15 mg CTec2/g glucan for 6 h.

Enzymatic hydrolysis of pretreated biomass is typically carried out under shaking/mixing conditions. To assess whether the observed changes in fiber length and width described earlier were influenced by the constant shaking/mixing applied during hydrolysis, the SPP200 R20 fiber fraction was hydrolyzed without shaking at an enzyme loading of 15 mg/g glucan. It was found that the extent of fiber fragmentation was decreased by about 60% when the reaction mixture was carried out without shaking/mixing (Figure 5), although similar hydrolysis yields were obtained (data not shown). Thus, it is possible that rather than the cellulases directly “cutting” the fibers, they might act on the dislocation regions within the pulp fibers which, under the shaking/mixing conditions typically used during enzymatic hydrolysis, results in the mechanically assisted fragmentation of the fibers.Typically, as the initial solids loading is increased, the time required for biomass deconstruction due to fiber fragmentation also increases. We next assessed whether the presence of sugars also decreased fiber fragmentation even when hydrolysis is carried out under shaking/mixing conditions. When the SPP200 R20 fiber fraction was hydrolyzed in the presence of 5% w/w glucose at an enzyme loading of 15 mg/g glucan, it was apparent that the addition of this supplementary glucose decreased the extent of fiber fragmentation by about 70% and reduced any observed decreases in fiber width (Figure 5). When the total amount of unbound enzymes was determined in the absence and presence of 5% w/w glucose using a modified ninhydrin assay, it was apparent that, of the total protein initially added to the reaction mixture, 72 ± 2% and 64.8 ± 0.5% were bound to the substrate, respectively. The addition of glucose resulted in about 9% less enzyme being bound to the substrate and it may, at least in part, have contributed to the decrease in the extent of fiber fragmentation (Figure 5). However, it is also possible that sugar inhibition of the bound enzymes might have contributed to the more limited fiber fragmentation that was observed.

During batch enzymatic hydrolysis of washed pretreated substrates, high concentrations of soluble sugars are not expected to be present at the initial stages of hydrolysis. Therefore, sugar inhibition of fiber fragmentation is likely to be negligible when hydrolysis is carried out in a batchwise fashion. However, during the batch enzymatic hydrolysis of unwashed pretreated substrates and whole slurry hydrolysis, a substantial amount of soluble sugars is already present at the early stages of hydrolysis. This could potentially inhibit fiber fragmentation and decrease the efficiency of hydrolysis, particularly by influencing the efficiency and time of biomass deconstruction at high initial solids loading.

## Conclusions

Steam and organosolv pretreated softwood and hardwood substrates were used to generate pulp fibers of varying fiber lengths and chemical composition. Only slight differences in fiber width were observed for all of the fiber fractions. Regardless of the pretreatment method and the nature and chemical composition of the fiber fraction or enzyme loading used, under typical hydrolysis conditions, the longer pulp fibers were rapidly fragmented during the early stage of hydrolysis (when cellulose hydrolysis yields were lower than 20%). Enzymatic fiber fragmentation was more apparent when the reaction was agitated, consequently enhancing the mechanically mediated fragmentation of the fibers. The major differences which could be detected in the pulp fiber characteristics throughout the course of hydrolysis were changes in the mean fiber width. It is possible that the observed changes were due to the simultaneous swelling and peeling/erosion of pulp fibers. It appears that, once particle size reduction reaches a constant size, the deconstruction of the fibers proceeds by predominant swelling and peeling/erosion modes of action rather than by fiber fragmentation.

## Methods

### Enzyme cocktail and total protein quantification

The cellulase preparation Cellic CTec2 (Novozymes North America Inc., Franklinton, NC) was used in all of the enzymatic hydrolysis experiments.

The total protein content of Cellic CTec2 (209.8 mg/mL) was determined using a modified ninhydrin protocol. Briefly, proteins in a diluted Cellic CTec2 solution and bovine serum albumin solutions in the range of 50 to 800 μg/mL were hydrolyzed with 6 M HCl for 90 min at 130°C. After neutralization with 50% w/v NaOH solution (Fisher) at room temperature, ninhydrin (2% nynhydrin reagent solution, Sigma) was added and allowed to react for 20 min at 100°C, followed by the addition of 50% (v/v) ethanol. The final reaction mixtures were transferred to a 96-well microtiter plate and color development was monitored at A_570nm_ using a multichannel ThermoMax Microplate Reader (Molecular Devices, Sunnyvale, CA). The Cellic CTec2 solution was analyzed with three repetitions, and bovine serum albumin samples were used as the protein standard.

### Lignocellulosic feedstocks and pretreatment

Softwood (Douglas fir and lodgepole pine) and hardwood (hybrid poplar) chips were pretreated by either SO_2_-catalyzed steam or ethanol-organosolv pretreatment under the conditions shown in Table 1 and as previously described [19,25]. All of the water-insoluble, cellulose-rich fractions were kept in refrigerated storage (4°C) until they were used for analysis and hydrolysis.

### Chemical analysis of pretreated substrates

The chemical composition of the pretreated materials was determined according to Technical Association of the Pulp and Paper Industry (TAPPI) standard method T222 om-88. Monomeric sugars were measured by high performance anion exchange chromatography with pulsed amperometric detection (HPAEC-PAD). A Dionex system with CarboPac PA1 column was used with fucose as internal standard, as previously described [19]. All of the analyses were performed in triplicate. The carbohydrate and lignin contents are described in Table 1.

### Fiber fractionation

Fiber populations that differ from one another in terms of initial fiber distributions to allow comparison with respect to their response to enzymatic hydrolysis were produced by sieving the pretreated materials in a set of sieves (20, 40, and 100 mesh) using a Bauer-McNett fiber classifier [26]. This equipment is commonly used in the pulp and paper industry for fractionating fiber slurries diluted in water. The major populations for each pretreated material were collected and classified as either R20 (fibers retained in sieve 20 mesh), R40 (fibers retained between the 20 and 40 mesh sieves), R100 (fibers retained between 40 and 100 mesh sieves), or F100 (fibers that passed through 100 mesh sieve) as listed in Table 2.

### Enzymatic hydrolysis

Batch hydrolysis was carried out in sodium acetate buffer (50 mM, pH 4.8) at 2% (w/w) solids loading. The reaction mixtures (1 mL) were mechanically shaken in an orbital shaker (Combi-D24 hybridization incubator) at 50°C. Cellic CTec2 was added at 15 or 30 mg protein/g glucan. Over a period of 72 h, samples were taken and heated at 100°C for 10 min, and centrifuged at 4,000 rpm for 15 min at room temperature. The supernatants were collected for quantification of glucose released, and the residual hydrolysis materials were retained for fiber analysis. All of the experiments were performed in triplicate.

The glucose concentration was determined using a microscale enzymatic assay involving glucose oxidase and horseradish peroxidase as adapted by Berlin et al. [27] The hydrolysis yields (%) of the pretreated substrates were calculated from the glucan content as a percentage of the theoretical glucan present in the substrate.

### Effect of shaking and soluble sugar on fiber properties

The effects of shaking and soluble sugar on the changes in fiber length and width during enzymatic hydrolysis were evaluated by carrying out enzymatic hydrolysis of the steam pretreated poplar fraction (SPP200 R20) at 15 mg/g glucan with and without shaking, and in the absence and presence of 5% (w/w) glucose. Other hydrolysis conditions and analyses were the same as described earlier.

### Fiber analysis

Changes in fiber dimensions (fiber length, fiber width), population, and distribution were determined using a high resolution Fiber Quality Analyzer (LDA02, OpTest Equipment, Inc., Hawkesbury, ON, Canada) as previously described [23]. The number of fibers counted per sample was 20,000. The ranges of fiber length and fiber width measured in this study were 0.05 to 10.00 mm and 7 to 60 μm, respectively. All samples were run in duplicate.

The following equation (Equation 1) was used to calculate the relative amount of short fibers over the course of hydrolysis for each sample:

$$\mathrm{RS}F_{t}=\left(\frac{\sum_{i=\mathrm{nc}}^{200}F_{i}}{\sum_{i=\mathrm{nc}}^{n}F_{i}}\right)\;{\left(\frac{\sum_{i=\mathrm{ic}}^{n}F_{i}}{\sum_{i=\mathrm{nc}}^{200}F_{i}}\right)}_{t=0}$$

where *RSF*_t_ is the relative amount of short fibers (from 50 to 200 nm in length) at a particular hydrolysis time *t*; *F*_i_ is the number of fibers of *i* length; *nc* is the low cut-off length for the particle size analyzer, *n* is the longest fiber length; and *t* is the hydrolysis time.

### Microscopy analysis

Scanning electron microscopy (SEM) was performed on the unhydrolyzed, size-fractionated pulp fibers to check for the presence of smaller particles. Prior to SEM analyses, samples were freeze-dried and then sputter-coated with 10 nm Au/Pd (80:20 mix) using a Cressington 208 HR High Resolution Sputter Coater (Watford, UK). Images were obtained using a Hitachi S-2600 N Variable Pressure SEM (VP-SEM, Toronto, ON, Canada). At least four samples from each sample were evaluated.

## Abbreviations

OPLP: organosolv pretreated lodgepole pine; OPLP R20: organosolv pretreated lodgepole pine pulp fibers retained in sieve 20 mesh; SPDF: steam pretreated Douglas fir; SPDF F100: steam pretreated Douglas fir pulp fibers that passed through 100 mesh sieve; SPDF R100: steam pretreated Douglas fir pulp fibers retained between 40 and 100 mesh sieves; SPP180: steam pretreated poplar at 180°C; SPP180 R20: steam pretreated poplar pulp fibers retained in sieve 20 mesh; SPP200: steam pretreated poplar at 180°C; SPP200 R20: steam pretreated poplar pulp fibers retained in 20 mesh sieve; SPP200 R40: steam pretreated poplar pulp fibers retained between the 20 and 40 mesh sieves.

## Competing interests

The authors declare that they have no competing interests.

## Authors’ contributions

VA conceptualized, designed, and carried out the experiments, analyzed the results, and drafted the manuscript. KG helped carry out some of the experiments and contributed to the draft of the manuscript. JS helped conceptualize, write, and review the manuscript. All authors have read and approved the final manuscript.
